# Association of food security and other socio-economic factors with dietary diversity and nutritional statuses of children aged 6-59 months in rural Bangladesh

**DOI:** 10.1371/journal.pone.0221929

**Published:** 2019-08-29

**Authors:** Nazia Binte Ali, Tazeen Tahsina, Dewan Md. Emdadul Hoque, Mohammad Mehedi Hasan, Afrin Iqbal, Tanvir M. Huda, Shams El Arifeen

**Affiliations:** Maternal and Child Health Division (MCHD), icddr,b, Dhaka, Bangladesh; Universidade de Sao Paulo, BRAZIL

## Abstract

**Introduction:**

Dietary diversity score (DDS) is a proxy indicator for measuring nutrient adequacy. In this study, we aimed to identify the nutritional statuses and current patterns of DDS among children between 6–59 months old and their associations with different individual and household level factors in rural Bangladesh.

**Methods:**

The Nobokoli programme of World Vision Bangladesh was implemented in Mymensingh, Sherpur, Rangpur, Dinajpur, Thakurgaon, Panchagar, and Nilphamari districts of Bangladesh between 2014 and 2017. A cross-sectional community household survey was administered between July and October 2014 to collect baseline data to evaluate the Nobokoli programme. A total of 6,468 children between 6–59 months old were included in the final analysis. Anthropometric data was collected following WHO guidelines on using wooden height and digital weight scales. We collected food intake information for the past 24 hours of the survey. The WHO’s child growth standard medians were used to identify the nutritional indices of stunting, wasting, and underweight. Food items consumed were categorized into nine food groups and the DDS was constructed by counting the consumption of food items across these groups during the preceding 24 hour period. The association of DDS and nutritional status (stunting, wasting and underweight) with sociodemographic factors and household food security status were examined using multivariable models; linear regression and logistics regression respectively.

**Results:**

The prevalence of stunting, wasting and underweight among children aged 6-59months were 36.8%, 18.2% and 37.7% respectively. Our findings revealed that almost all children ate any form of starch followed by consumption of milk or milk products (76%) and fleshy meat /fish (61%) respectively. The mean DDS among children was 3.93(sd 1.47). Forty percent of the children obtained a DDS score less than 4. Multivariable analysis suggested that children whose mothers had higher educational attainment and are skilled workers had higher DDS (15% and 48% respectively) compared to their counterparts. The DDS showed strong positive association with household wealth status. Children from food secure households had 26% higher DDS compared to children from food insecure households. Similarly, increasing maternal education and household wealth were found to be protective against childhood stunting and undernutrition.

**Discussion:**

Our findings reiterate the need for improving household socioeconomic factors and household food security status for improving dietary diversity practices and nutritional status of children. Evidence-based solutions are needed to be implemented and expanded at scale to ensure appropriate dietary practices and improve nutritional status of the children in local context.

## Introduction

Adequate nutrition is a prerequisite for optimal growth and cognitive development of children. Undernourished children have higher risks of mortality and morbidity [1]. Globally, undernutrition contributes to approximately 3.2 million children’s deaths annually and leads to an 11% reduction in disability adjusted life year (DALY), with the majority of the burden coming from low and middle income countries (LMIC) [2]. Bangladesh is one of the LMICs with high prevalence of childhood undernutrition. In 2014, around 36% and 14% of under-five children in Bangladesh were stunted and wasted respectively [3].

The factors influencing childhood undernutrition are multifaceted [4]. The UNICEF conceptual framework of Malnutrition identified improper feeding practices as an immediate cause of childhood undernutrition [5]. Infant and young child feeding (IYCF) practices in Bangladesh is not satisfactory [6]. In 2014, only 23 percent of children between the ages 6 to 23 months were fed appropriately according to recommended IYCF practices [3]. Ensuring a minimum standard of food diversity, food quantity and food frequency still remains as an concern here[6–9]. At the household level, household food insecurity (HFI) status, socioeconomic conditions, and knowledge regarding nutritious foods are some of the key underlying factors influencing the nutritional status of children [9, 10]. Approximately 34% rural households in Bangladesh suffered from food insecurity in 2013 [3, 11, 12]. Ensuring household food security is thus among the most important long term factors that could prevent childhood under nutrition [13–15].

Dietary diversity is one way of conceptualising dietary adequacy and optimal nutrient intake. Several studies in both developed and developing countries have linked dietary diversity indicators with improved nutrient intake[16]. Evidence from 11 demographic surveys showed positive association of dietary diversity with sociodemographic factors when controlling for other individual, maternal, household, and community-level factors [17, 18]. Generally children from poor households had lower dietary diversity as their meals are based predominantly on starches with little fish, meat, egg, vegetables, and fruits[17]. Maternal education is another significant predictor of dietary practices of children who are between 6–23 months old[17]. While the association between sociodemographic factors and dietary practices has been established in different settings, several studies have suggested that the association of dietary diversity with structural factors like food security in local context are needed[17, 19].

Although first 1000 days of life is considered as “Golden Period” for nutritional intervention, ensuring optimal nutrition in the first five-years is important for cognitive development. Children under five years are especially vulnerable to undernutrition and burden of undernutrition is higher in the rural areas compared to the burden in urban areas due to inequity in availability and accessibility of food items. Majority of the studies exploring the determinants of dietary diversity and nutritional status of children examined the association among children aged less than 2 years of age [7, 20–23] However, there is limited available evidence on the association between household food security status and dietary diversity among children under five of age in rural Bangladesh. In this study, we aimed to explore the nutritional statuses and current patterns of DDS among children aged 6–59 months and their associations with various immediate and underlying individual and household level factors including household food security status. Identifying this relationship will help policy makers identify vulnerable groups in a community and develop targeted intervention programmes.

## Materials and methods

### Study design and data

World Vision Bangladesh implemented “Nobokoli,” an integrated nutrition programme, in 20 low performing sub-districts of Mymensingh, Sherpur, Rangpur, Dinajpur, Thakurgaon, Panchagar, and Nilphamari districts in Bangladesh from 2014 to 2017. Nobokoli interventions targeted nutrition, water, sanitation, hygiene, and livelihood to address the underlying causes of childhood undernutrition. A large community based household survey was conducted as part of baseline assessment of Nobokoli programme evaluation. The cross-sectional baseline data had utility beyond the purpose of program evaluation and was used to provide insight on the nutritional statuses of children for this study.

Mothers of under five children were interviewed during baseline assessment between July to October 2014. The interviews provided information on socio-demographic characteristics, household food security status, feeding practices of the children, childhood illnesses, care seeking practices etc. A separate team of data collectors conducted anthropometric measurements of the children whose mother’s were interviewed.

### Sampling and sample size

This study used a subset of data from a quasi-experimental study to evaluate the Nobokoli program. The sample size was calculated using the formula: c = 1+ (zα/2+zβ) 2 [π0 (1-π0)/n+π1(1-π1)/n + k 2(π02 +π12)]/ (π0 -π1)2 (Hayes, 1999). Here, k is the coefficient of variation between the clusters within each arm. We calculated sample size for several indicators such as stunting, wasting, underweight among under-five children, exclusive breast feeding practices, IYCF practices etc. Using the coefficient of variation (k) between the range of 0.074 for anaemia among under-5 children to 0.423 for delivery-assisted by skilled personnel[24, 25], we estimated that highest 70 clusters were needed in each arm to detect 20% change in selected indicators with 80% power, 5% level of significance and 10% rate of refusal. The highest required sample per cluster was 52 which yield a total of 7280 under five children samples for the survey.

A multistage cluster sampling technique was used to select study participants. Sub-districts were defined as strata and villages with a population between 100–1800 were defined as clusters. Villages with smaller populations were combined to form clusters. In the first stage, Probability Proportional to Size (PPS) sampling was used to select 15 sub-districts of 20 Nobokoli sub-districts. In the second stage, 140 clusters were selected from the 15 intervention and comparison sub-districts using PPS. In the final stage of sampling, 52 mothers of children under the age of 5 were selected from each cluster for interviews. Overall, 8,679 households were surveyed and the response rate was 99.9%. Details of the baseline survey has been published elsewhere [26, 27].

**Fig 1:**
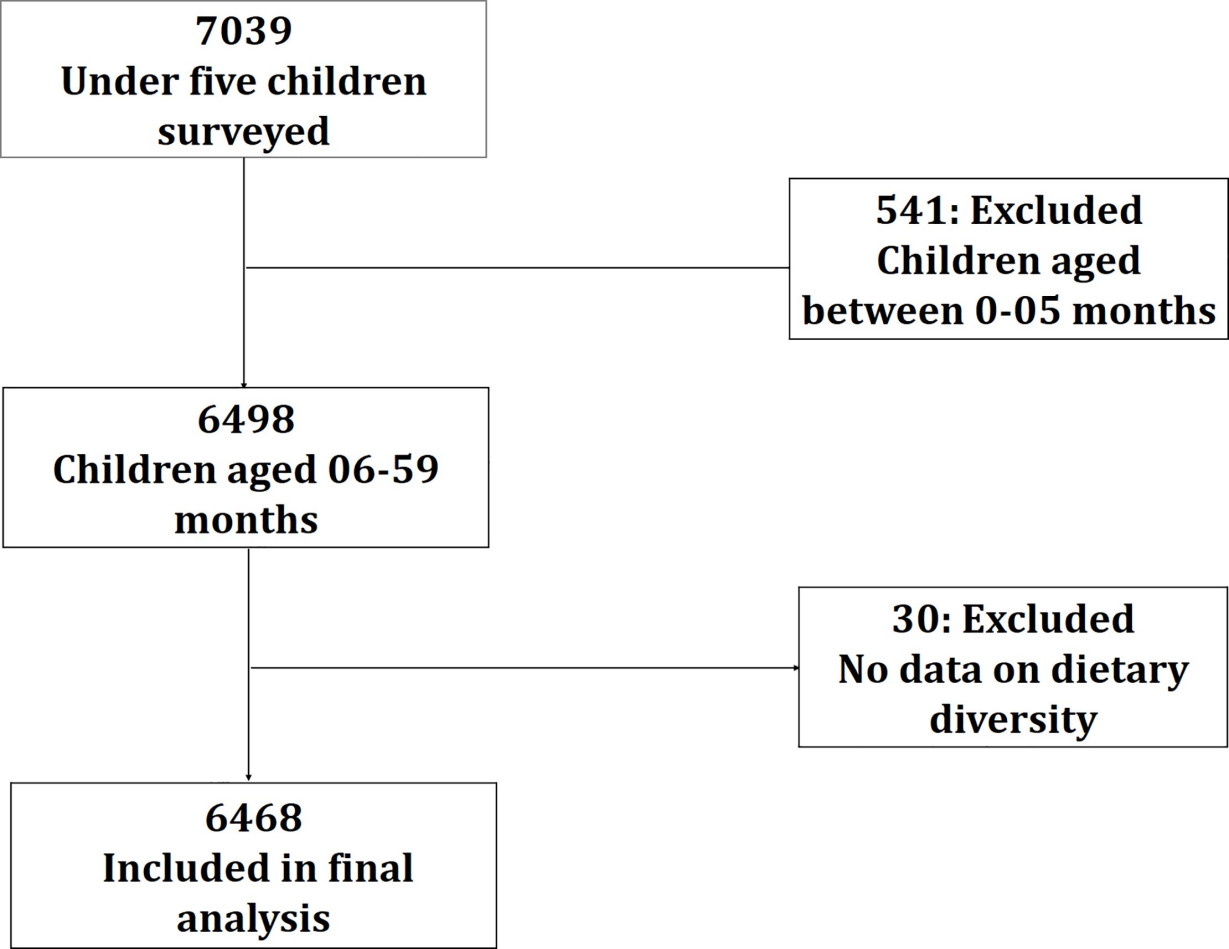
Participant flow diagram.

A total of 7039 mothers took part in the baseline survey. Children were omitted from the study population if complete information on feeding practices were not available. The final study population included 6468 children between 6 and 59 months old. Details of the participant flow in this analysis is shown in Fig 1.

### Ethical consideration

The study was reviewed and approved by institutional review board of icddr,b. Approved protocol number was PR#14044. We followed the “Good Clinical Practices” guideline by International Conference on Harmonisation (ICH) expert working group for data collection. Participation in the survey was voluntary. Informed written consents were taken from the study respondents before interview. In case of illiterate participant, thumb print was taken as part of informed consent.

### Measurement process & quality assurance

A pre-validated structured questionnaire in Bangla was used for data collection. Mothers of the under-five children were interviewed after obtaining informed written consent. Experienced and trained data collectors conducted the household interviews. Anthropometric measurements were collected following WHO Anthro manual. Wooden height scales were used to measure height and TANITA digital weight scales were to measure weight. Anthropometric tools were calibrated before each day data collection. We collected three measurements of height and weight of each individual to ensure consistency. Data collection activities were monitored by two tiers of field supervisors who randomly selected interviews for direct observation. All completed questionnaires were double checked for completeness and consistency. Five percent of women were re-interviewed by a separate data quality assurance team to ensure data captured were accurate.

### Variables

The primary outcomes of interest were the nutritional statuses and Dietary Diversity Score(DDS) of food intake in children aged 6–59 months. WHO Child Growth Standard Medians were used for identify whether a child was stunted, wasted, or underweight. Children with a height for age z score two standard deviations or below the standard median were identified as stunted[28]. Children with a weight for height z score two standard deviations or below standard median were considered wasted and those with weight for age z scores two standard deviations or below the standard median were considered underweight[28].

In low resource settings, DDS is a simple, valid, reliable, and commonly documented indicator to measure the nutrient adequacy of children [29–32]. There are different approaches to estimating DDS which include simple food counts, simple food group counts, or weight based food group counts [29, 33]. The study used a simple food count DDS score due as this was the most appropriate method given the limited resources for the study[30, 32–34]. Mothers were asked about the food intake of their children in the past 24 hours, using the standard coded WHO IYCF questionnaire. The food items reported were categorised into nine food groups: starchy staples, milk/milk product, egg, fleshy meat/fish, organ meat, legumes and nuts, vitamin A rich fruit and vegetables, dark green vegetables, and other fruits and vegetables[12, 35]. Individual DDS were calculated by adding the number of food groups consumed by the child in the past 24 hours [21, 32].

Explanatory variables for this study included household socioeconomic and food security statuses. Other characteristics examined in this study were mothers’ ages, educational attainments, and employment statuses. Mother's age data was collected in years and two categorical age groups were created; 15–24 years and 25–49 years. Educational attainment only considered formal schooling and was divided into three categories; no education, primary incomplete (1–4 years of education) and primary and above (5 or more years of education). Employment status fell into one of three different categories: homemakers, unskilled workers, and skilled workers.

Information on the household’s residence type, usual source of drinking water, access to sanitary latrines, ownership of homestead and other lands, ownership of household assets, possession of domestic animals, and transportation means were collected. Principal component analysis (PCA) was used to construct an asset score. These scores were then divided into five wealth quintiles. Information on a household’s food expenditure in the month preceding the survey was collected in taka. Monthly food expenditure data was not adjusted for household’s consuming self-produced foods resulting in potential underestimation of the food expenditure by each household; one of the limitation of this variable.

The Food and Agriculture Organization (FAO) defined household food insecurity as a state or condition in which people experienced limited or uncertain physical and economic access to safe, sufficient, and nutritious food to meet their dietary needs or food preferences[36]. Food insecurity has four domains; availability, accessibility, utilization, and sustainability. In this study, we explored the accessibility domain of food insecurity. The standardized “Household Food Insecurity Access Scale” questionnaire developed by Food And Nutrition Technical Assistance (FANTA) initiative of USAID was used to measure household food security status[26]. For our analysis, we created a binary variable for household food security status: food secure and food insecure.

### Data analysis

All statistical analysis were done using Stata 13. We examined the prevalence of stunted, wasted, and underweight children. The proportion of children consuming foods from different food groups and the distribution and variations in mean DDS across different socio-economic and household food security status were explored. Existing literature as well as bivariate analysis guided the selection explanatory variables. The Kruskal Wallis test was performed to test the association between DDS with categorical explanatory variables: sex of the children, mother's age, education, occupation, household's monthly expenditure on food, food security status and wealth quintiles. Variables found to produce significant differences in DDS then selected for multivariate linear regression in a stepwise fashion. Socio-economic determinants of childhood stunting, wasting and underweight were examined using three separate multivariable logistic regression models. The unit of analysis was the individual. We tested all underlying assumptions of multivariable regressions. We used the Variance Inflation Factor (VIF) to assess multi-collinearity. Mean VIF of the explanatory variables in the multivariable models were <4 which ensures absence of multi-collinearity. Association of the explanatory variables with DDS were reported using coefficients whereas odd ratios were reported for determinants of stunting, wasting and underweight. P-values <0.05 demonstrated statistically significant association.

## Results

Table 1 summarises socioeconomic characteristics of the household with children aged 6–59 months. Fifty-two percent of the sampled children were boys. The majority of mothers (63%) were between the ages of 25–49. Nineteen percent of the mothers received no formal education and majority of the mothers were homemakers (90%). Sixty six percent of the households spend BDT 5000 / USD 60 or less on food per month. 43% of households experienced food insecurity. Households were equally distributed across wealth quintiles.

**Table 1:** Socioeconomic characteristics of households having children aged 06–59 months in rural areas of Bangladesh, 2014.

Background characteristics		n = 6468	Percentage (%)
**Characteristics of children**			
Sex	Boy	3,367	52.1
	Girl	3,101	47.9
**Characteristics of Mothers**			
Mothers Age	15–24 years	2,380	36.8
	25–49 years	4,088	63.2
Mother’s Education	No education (0 years)	1,247	19.3
	Primary incomplete (1–4 years)	1,172	18.1
	Primary to secondary (5+ years)	4,049	62.6
Mother’s Employment Status	Homemaker	5,819	90.0
	Unskilled worker	300	4.6
	Skilled worker	349	5.4
**Household Characteristics**			
Household’s monthly expenditure on food(in taka)	≤ BDT 5,000 / USD 60	4,281	66.2
	> BDT 5,000 / USD 60	2,126	32.9
	Missing	61	0.9
Households food security status	Food secure	3,673	56.8
	Food insecure	2,795	43.2
	Mild Food Insecure	833	12.8
	Moderate Food Insecure	1,417	21.9
	Severe Food Insecure	545	8.4
Wealth Quintile	Lowest	1,327	20.5
	Second	1,302	20.1
	Middle	1,299	20.1
	Fourth	1,277	19.7
	Highest	1,263	19.5

**Fig 2:**
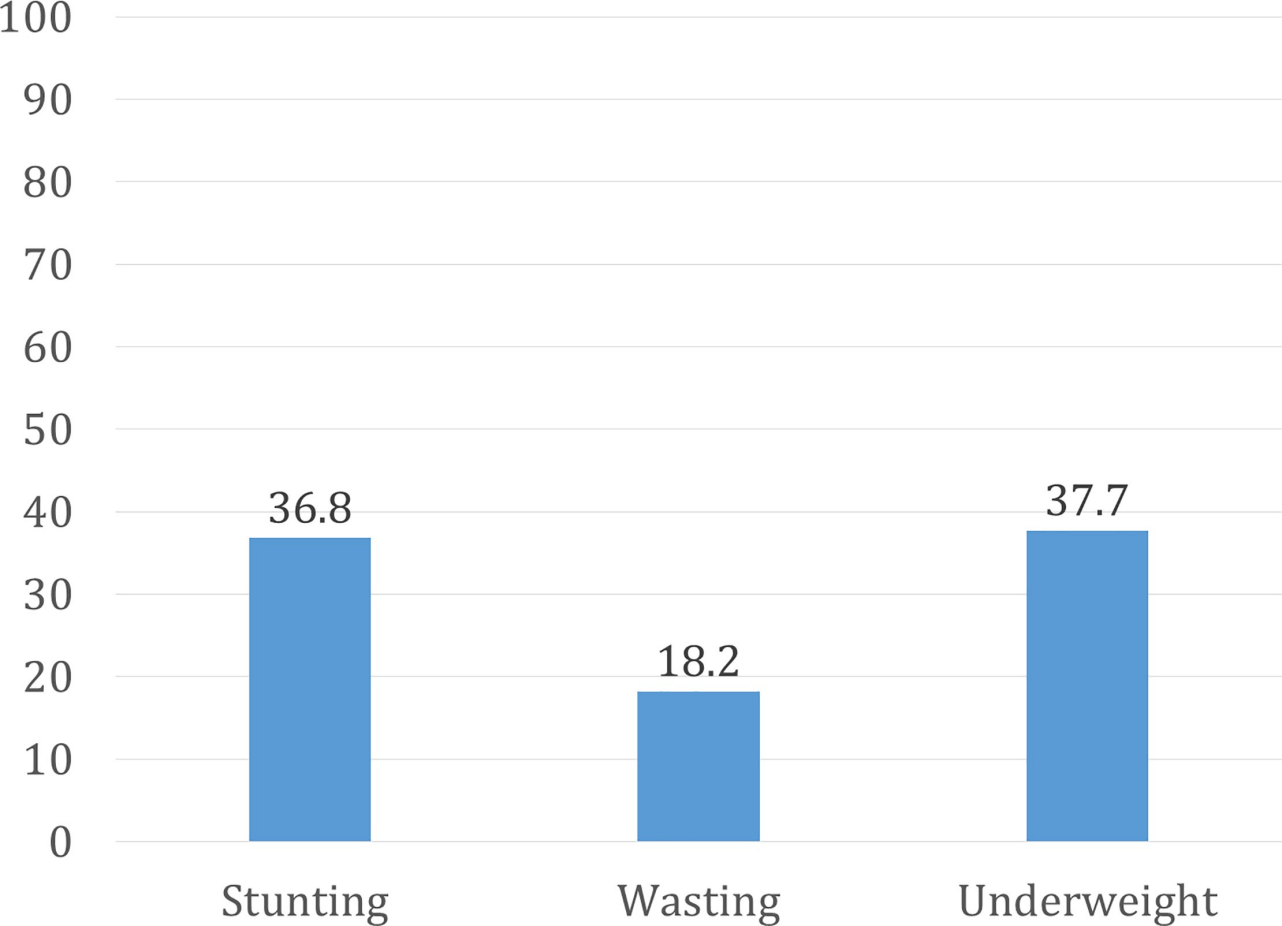
Prevalence of stunting, wasting and underweight among children aged 6-59months in rural areas of Bangladesh, 2014.

Fig 2 displays the prevalence of stunting, wasting, and underweight statuses among children in the study population with 36.8%, 18.2% and 37.7% of children belonging to each respective category.

**Fig 3:**
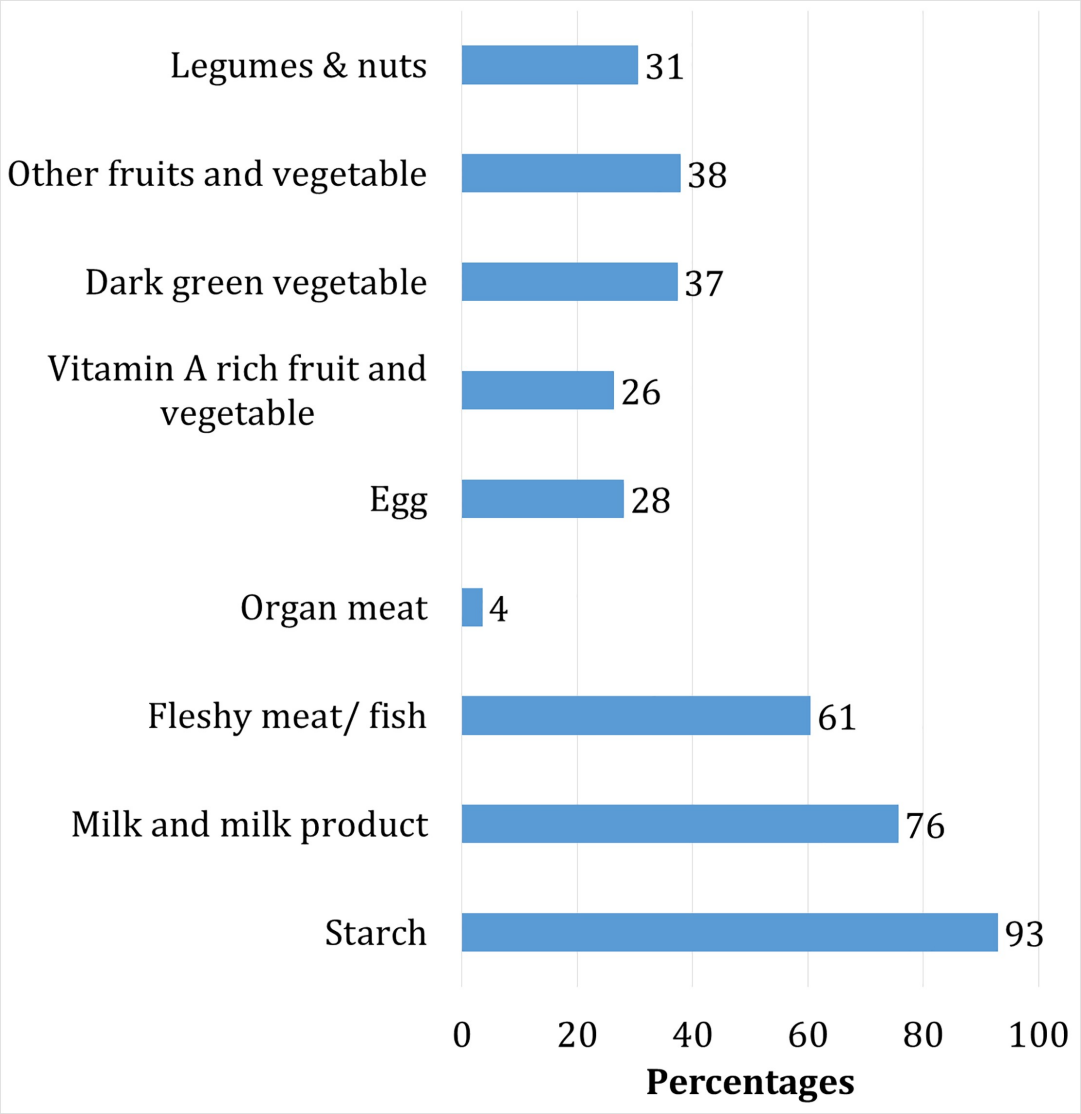
Proportion of children aged 6–59 months consumed foods from different food groups in the past 24 hours of survey in rural areas of Bangladesh, 2014.

**Fig 4:**
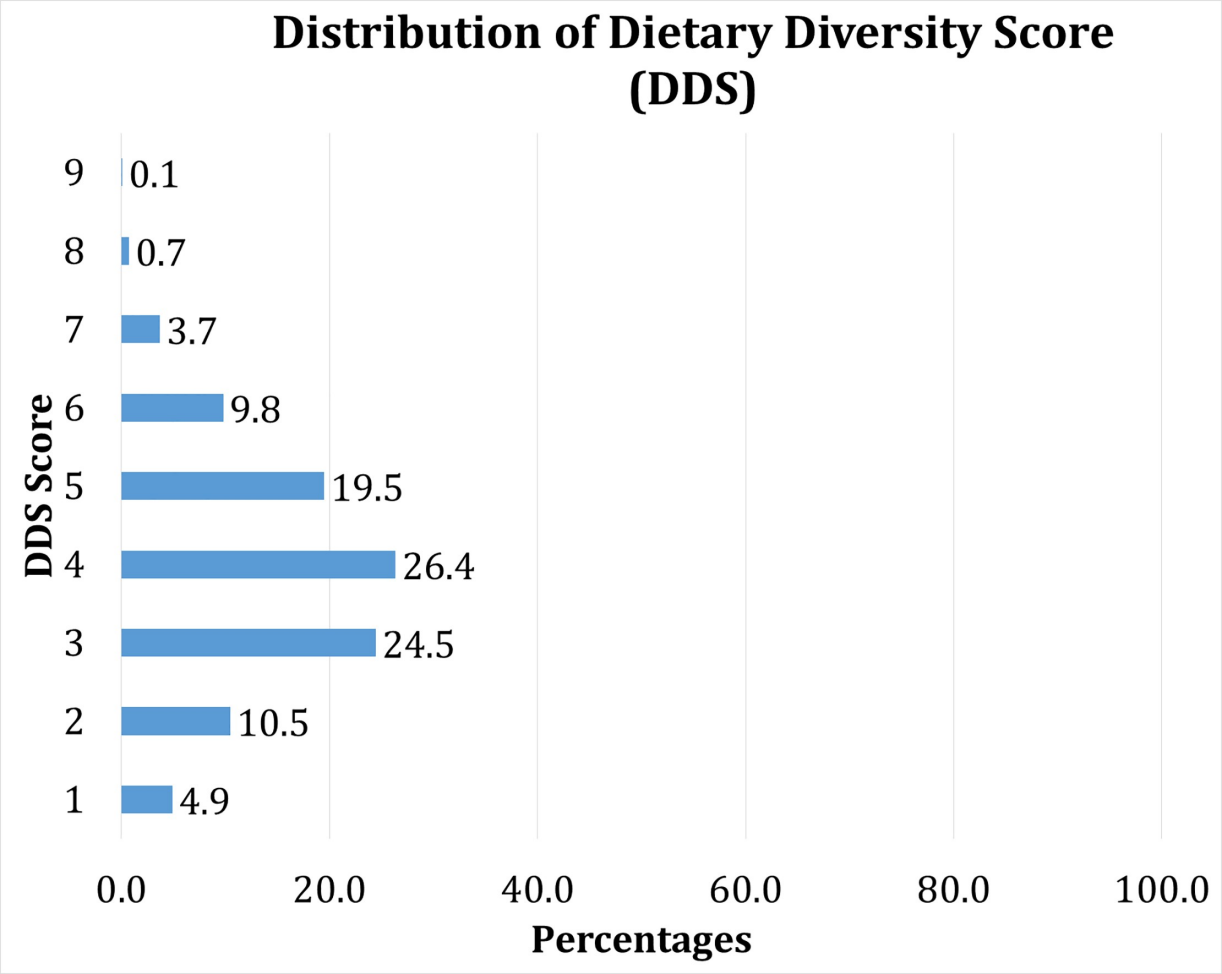
Distribution of Dietary Diversity Score (DDS) among children aged 6-59months in rural areas of Bangladesh, 2014.

Fig 3 represents the patterns of consuming foods from different groups. The data revealed that food groups with the highest levels of consumption in the 24 hours period preceding the survey were starchy staples, followed by milk/milk products (76%), and animal proteins (65%). Thirty-seven percent of the children ate dark green vegetables while 28% of consumed eggs in 24 hour recall period. DDS scores greater than 4 indicate adequate nutrient intake, however 40% of children in this study had scores less than 4(Fig 4).

The mean DDS among sampled children was 3.93 (sd 1.47). DDS did not vary across the sex of the children or their mothers’ ages (Table 2). Children whose mothers had higher educational attainment and were skilled workers, had a significantly higher mean DDS. The mean DDS increased as household wealth and monthly expenditure on food increased. Children whose households did not face food insecurity were found to have a higher mean DDS when compared to their counterparts (Table 2).

**Table 2:** Mean Dietary Diversity Scores (DDS) among children aged 6–59 months across different socioeconomic characteristics in rural areas of Bangladesh, 2014.

Background characteristics		Number of children	Mean DDS (sd)	P values from (kwallis tests)
**Mean DDS**		**6,468**	**3.93 (sd 1.47)**	
**Characteristics of children**				
Sex	Boy	3,367	3.93 (sd 1.44)	0.709
	Girl	3,101	3.93 (sd 1.49)	
**Characteristics of Mothers**				
Mothers Age	15–24 years	2,380	3.93 (sd 1.46)	0.792
	25–49 years	4,088	3.93 (sd 1.47)	
Mother’s Education	No education (0 years)	1,247	3.68 (sd 1.37)	<0.000
	Primary incomplete (1–4 years)	1,172	3.74 (sd 1.41)	
	Primary to secondary (5+ years)	4,049	4.06 (sd 1.49)	
Mother’s Employment Status	Homemaker	5,819	3.90 (sd 1.46)	<0.000
	Unskilled worker	300	3.85 (sd 1.40)	
	Skilled worker	349	4.49 (sd 1.52)	
**Household Characteristics**				
Households monthly expenditure on food(in taka)	≤ BDT 5,000/ USD 60	4,281	3.79 (sd 1.40)	<0.000
	> BDT 5,000/ USD 60	2,126	4.20 (sd 1.54)	
Households food security status	Food Insecure	2,795	3.69 (sd 1.38)	<0.000
	Food secure	3,673	4.12 (sd 1.50)	
Wealth Quintile	Lowest	1,327	3.58 (sd 1.32)	<0.000
	Second	1,302	3.77 (sd 1.36)	
	Middle	1,299	3.88 (sd 1.43)	
	Fourth	1,277	3.99 (sd 1.52)	
	Highest	1,263	4.46 (sd 1.55)	

In the next stage, we looked at the determinants of DDS among children aged 6-59months. Table 3 presents the adjusted coefficients and p values of DDS from a multivariable regression analysis which used socioeconomic predictors. Children of mothers with primary or higher educations had a 15% higher DDS when compared to children of mothers with no education. Simlarly, on average children of mothers who were skilled workers had 47% higher DDS than children whose mothers were homemakers. Household’s economic status showed strong positive association with DDS among the children. Children from households spending BDT 5000/ 60USD or more on food monthly, had 26% higher DDS when compared to their counterparts. DDS also showed a dose response relationship with household wealth. The children from the highest wealth quintile had 54% higher DDS than the children from the lowest wealth quintile. Children from the food secure households had 21% higher DDS when compared with children from food insecure households.

**Table 3:** Association of food security and socioeconomic factors with Dietary Diversity Score (DDS) among children aged 6-59months in rural areas of Bangladesh, 2014.

Background characteristics		Unadjusted		Adjusted	
		Coefficients	P values	Coefficients	P values
**Characteristics of children**					
Sex	Boy	Ref		Ref	
	Girl	-0.00	0.942	-0.00	0.940
**Characteristics of Mothers**					
Mothers Age	15–24 years	Ref		Ref	
	25–49 years	0.00	0.956	0.00	0.903
Mother’s Education	No education (0 years)	Ref		Ref	
	Primary incomplete (1–4 years)	0.06	0.279	0.02	0.718
	Primary to secondary (5+ years)	0.38	<0.000	0.15	<0.05
Mother’s Employment Status	Homemaker	Ref		Ref	
	Unskilled worker	-0.05	0.531	0.03	0.736
	Skilled worker	0.59	<0.000	0.48	<0.000
**Household Characteristics**					
Households monthly expenditure on food(in taka)	≤ BDT 5,000/ USD 60	Ref		Ref	
	> BDT 5,000/ USD 60	0.41	<0.000	0.26	<0.000
Households food security status	Food insecure	Ref		Ref	
	Food secure	0.42	<0.000	0.21	<0.000
Wealth Quintile	Lowest	Ref		Ref	
	Second	0.19	<0.001	0.09	<0.113
	Middle	0.29	<0.000	0.18	<0.05
	Fourth	0.40	<0.000	0.23	<0.000
	Highest	0.87	<0.000	0.54	<0.000

In addition to DDS scores, the socio-economic determinants of nutritional statuses were examined. Results from the multivariable analysis showed that the adjusted odds of being stunted or underweight decreased as maternal education and household wealth increased (Table 4).

**Table 4:** Association of food security and socioeconomic factors with nutritional status of the children aged 6-59months in rural areas of Bangladesh, 2014.

Background characteristics		Stunting		Wasting		Underweight	
		Adjusted		Adjusted		Adjusted	
		Odd ratios	P values	Odd ratios	P values	Odd ratios	P values
**Characteristics of children**							
Sex	Boy	Ref		Ref		Ref	
	Girl	0.98	0.814	0.96	0.511	1.06	0.240
**Characteristics of Mothers**							
Mothers Age	15–24 years	Ref		Ref		Ref	
	25–49 years	0.99	0.964	1.06	0.419	1.08	0.186
Mother’s Education	No education (0 years)	Ref		Ref		Ref	
	Primary incomplete (1–4 years)	0.80	<0.05	1.03	0.754	0.86	0.089
	Primary to secondary (5+ years)	0.63	<0.000	0.95	0.585	0.74	<0.000
Mother’s Employment Status	Homemaker	Ref		Ref		Ref	
	Unskilled worker	1.05	0.689	0.80	0.193	0.91	0.481
	Skilled worker	1.12	0.334	0.98	0.890	0.98	0.908
**Household Characteristics**							
Households monthly expenditure on food(in taka)	≤ BDT 5,000/ USD 60	Ref		Ref		Ref	
	> BDT 5,000/ USD 60	0.92	0.167	0.98	0.842	0.96	0.525
Households food security status	Food insecure	Ref		Ref		Ref	
	Food secure	0.94	0.314	0.95	0.462	0.95	0.414
Wealth Quintile	Lowest	Ref		Ref		Ref	
	Second	0.78	<0.05	0.92	0.446	0.75	<0.000
	Middle	0.87	0.105	0.96	0.659	0.87	0.098
	Fourth	0.83	<0.05	0.92	0.443	0.81	<0.05
	Highest	0.64	<0.000	0.76	<0.05	0.50	<0.000

## Discussion

In this study, we explored the nutritional statuses of children and the diversity of their food consumption. Bangladesh has burden of stunting, wasting and underweight among children 6–59 months in rural areas. Almost every child consumed a starchy staple in the 24 hours preceding the survey. A large majority of children consumed milk products (76%) and animal proteins (66%) during this period of time, while the consumptions of eggs, fruits, and vegetables were low. The mean DDS among the children sampled was 3.93 (sd 1.47) consistent with findings from other studies [37, 38]. Around 40% of the sampled children had a DDS less than 4 which indicates insufficient dietary diversity. Our findings also showed a strong positive association between DDS and maternal education, employment, and other socioeconomic factors such as household monthly expenditure on food, household wealth status and household food security status. Finally, increasing maternal education and household wealth were found to be protective against childhood undernutrition.

The prevalence of stunting, wasting and underweight among children 6–59 months were 36.8%, 18.2% and 37.7% respectively in the study areas. The findings were consistent with the prevalence reported in Bangladesh demographic and Health survey 2014[3]. The WHO defines a 30% or greater prevalence of stunting, a 15% of greater prevalence of wasting, and a 30% of greater prevalence of underweight children as warranting public health concern[28]. Underlying factors of childhood undernutrition are multi-dimensional. Poor feeding practices, specifically a lack of dietary diversity, is one of the immediate cause leading to such high prevalence of childhood undernutrition among children in Bangladesh [5].

Our study revealed that children from the food secure households had higher dietary diversity compared to the children from the food insecure households during the monsoon season, consistent with other findings [39, 40]. Poverty, low agricultural production, high food price, natural calamity and seasonality are some of the reasons behind household food insecurity in the developing countries[41–43]. Studies looking at the impact of seasonality on household food insecurity and nutritional status of children in Bangladesh confirms that the prevalence of food insecurity and growth faltering among children is higher during monsoon season compared to dry season [38, 44]. Initiation and expansion of new social safety net programmes such as cash transfer, food transfer and other income generating programmes can improve the capacity of people to acquire foods and thereby maintain and ensure household food security status during low harvest seasons [45–49].

Household food security is also related with agricultural production [42, 50–53]. Agricultural interventions can improve household food security status by increasing the availability of and accessibility to various nutritious food items. A study from Nepal suggests that each additional food group produced by the household increases the log odds of meeting minimum dietary diversity of children aged 18–24 month by 0.25 (p value 0.01)[54]. Intake of dairy, poultry, vitamin A rich fruits & vegetables & other vegetables improve with diversification of household food production [54].

Children from wealthier households have improved dietary diversity and lower levels of undernutrition. Wealthier households often use additional income to purchase non-staple foods, thus increasing household dietary diversity. A recent analysis found income to be a significant determinant of household dietary diversity in Bangladesh [55, 56]. Evidence from different demographic studies also indicated that families with higher income and more resources tend to have more diverse diets and lower levels of childhood undernutrition [17, 45, 57].

Educated mothers are more likely to have better knowledge and practices of nutritious food habits. Our study also linked mothers’ educational attainments with higher mean DDS and lower prevalence’s of undernutrition in children. These results are supported by findings from other developing countries [22, 56, 58]. Nutritional education programmes can improve dietary diversity practices and nutritional status of children [59–61]. Home based nutrition counselling improved diet quality of children from both food secure and insecure population [60–64]. However, the translation of education to improved dietary practices and subsequent improvements in nutritional status, is a lengthy process. Improvements in IYCF practices took as long as 18 months of counselling sessions by health staffs including regular home visits and growth monitoring in Peru[65, 66].

Culturally shaped dietary habits can act as bottlenecks and prevent dietary diversity and improved nutritional statuses of children. In Bangladesh, the major portion of regular meals consists of starchy staples. Also, women and children receive small share of food due to inequity in intra-household food distribution [16]. However, the scenario can be different for mother’s employed as skilled workers. The findings of this study confirmed those of others that children whose mothers are skilled workers are more likely to have higher DDS when compared to children with stay at home mothers [67, 68]. A working mother may have more control on food choices due to increased accessibility and affordability. They are also likely to have better knowledge about health and nutrition of their children, which may influence better feeding practices[49, 53, 68–72]. On a different note, given the nature of the work the women are involved in, further explorative study would be pertinent to identify the factors facilitating good feeding practices among working mothers in different areas.

### Strengths and limitations

The large geographically representative sample with a high response rate and the use of rigorous statistical analysis techniques have strengthened the findings of this study. Additionally, the usage of a well validated structured questionnaire may have controlled for instrumental and inter-rater biases. The use of a 24-hour food consumption recall period may have reduced the chance of recall bias when compared to longer recall periods. The minimized recall bias comes at the cost of providing no information on seasonal variations in food consumption and their usual food habits.

Some of the sampled children included in this study were breastfed at the time of this survey. Mothers’ dietary practices might have influenced the nutritional statuses of breastfed children. Failure to explore the association of mothers’ diets with their children’s’ nutritional statuses could have limited the findings of the study and requires further exploration in the subsequent studies. In addition, the cross-sectional design of the survey limits the use of study findings for causal inference. Further studies involving prospective monitoring of food security and dietary diversity determinants using 7 days or 3 month recall period questionnaires are required to establish epidemiological causality.

## Conclusion and recommendation

Bangladesh has high burden of childhood undernutrition. Ensuring dietary diversity for under-five children is a challenge here due to presence of high level of food insecurity. The 2013 Lancet Maternal and Child Nutrition series proposed a framework for prioritizing evidence based interventions to improve the dietary practices and nutritional statuses of children. The Lancet series identified behaviour change communication to improve complementary feeding practices, as a nutrition specific intervention, and investments in rural infrastructure, measures to increase agricultural productivity, the usage of consumer food subsidies and women’s education are some as nutrition sensitive interventions effective in addressing childhood nutrition. [2, 73, 74]. Our findings re-emphasized the need to combine knowledge with social transfers [75]. This study will build upon the available evidence on food security and other sociodemographic determinants of dietary diversity and childhood undernutrition. Findings from this study will also help policy makers identify high risk groups for childhood undernutrition and use context- specific solutions to ensure optimal dietary diversity in rural children between ages 6–59 months in Bangladesh.
